# Immersive virtual reality for learning about ecosystems: effect of two signaling levels and feedback on action decisions

**DOI:** 10.3389/fpsyg.2024.1359071

**Published:** 2024-04-24

**Authors:** Laurie Porte, Jean-Michel Boucheix, Louis Rapet, Véronique Drai-Zerbib, Jean-Luc Martinez

**Affiliations:** ^1^LEAD-CNRS, University of Bourgogne, Dijon, France; ^2^Arts et Métiers, Institute of Technology, LISPEN, HESAM University, Chalon sur Saône, France

**Keywords:** learning, virtual reality, visual signaling, feedback, cognitive processes, action decision making, presence, climate change education

## Abstract

**Introduction:**

The goal of the present study was to test the effect of signaling associated with feed-back in learning forest ecosystems in the context of realistic living forest simulator, in IVR conditions for students in agriculture. Two signaling modalities, corresponding to two signaling levels, were investigated: visual *flashing* of forest elements (tree species, plants, flowers, fungi, wet-areas etc.) and *marker-stones*, both with text in pop-up windows, in a 2x2 experimental plan.

**Methods:**

Ninety-three pupils of an agricultural technological high school had to explore (including physically), interrogate (search for) and select (using the joysticks) relevant elements of the forest in three living forest areas (visually delimited inside of a broader forest area) in order to choose (and justify) the best area, among the three, in which an equipped public-tourist reception site (picnic, resting, reception site) could be built. The chosen site must have the least possible negative impact on the ecosystem of the forest and its development over time. After their decision (and justification) they were provided a feed-back with a series of VR desktop multimedia slides showing the effect of this choice on the ecosystem of the chosen area. After the feed-back they had to decide and justify again whether they would change or maintain their first decision. Finally, subjective scales were also used in order to investigate presence, cognitive complexity, sickness and overall enjoyment.

**Results and discussion:**

Results showed significant positive effects of both signaling levels, and of the feed-back on the correct decision answers. Further, the combination, and interaction, between signaling and feedback seemed to enhance, the activation and retrieval from memory, of the task-relevant concepts. In addition, the results indicated a significant positive effect (medium size) of presence on decision performances, a finding which is consistent with the immersion principle.

## 1 Introduction and goal

**Figure 1:**
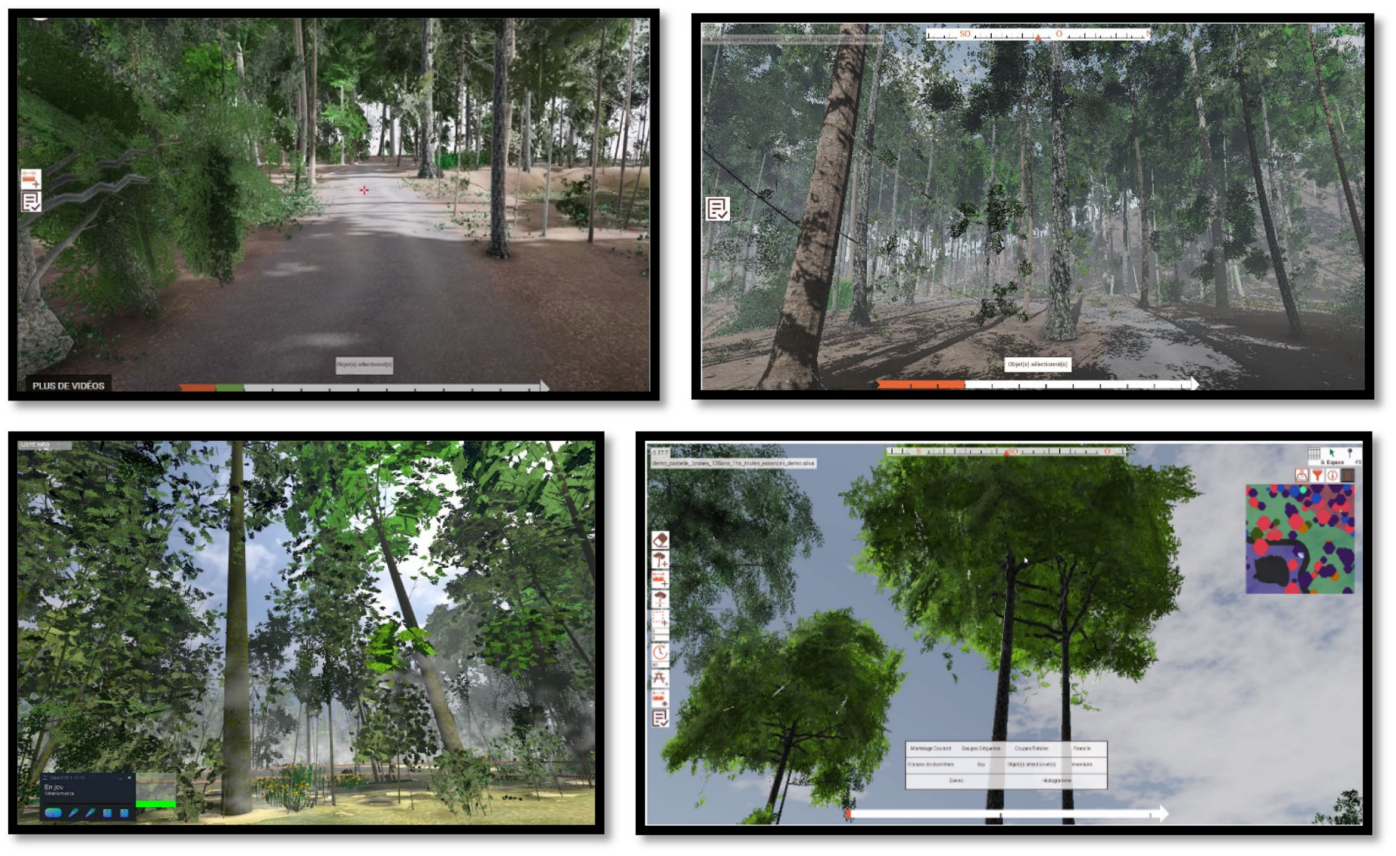
Snapshots of the VR simulation environment, from left to right and top to bottom: access path to the forest parcels, inside the parcels, view of the trees when the learner looks up toward the sky.

In a context of major environmental change, the use of immersive virtual reality (IVR) for the learning of complex ecosystems such as those found in forests could be a promising avenue to explore in the light of the ongoing changes in the training of students of agricultural forestry. “Seeing” the visible and non-visible forest elements and their interrelations in a 3D environment, visually observing, via IVR simulation of forest development, the potential “real” short-to-medium and long-term effect of human actions and forestry management decisions on tree species and the development of wildlife ecosystems could enhance learning and interest and improve professional decision-making (Figure 1). For example, a typical learning or training task performed by forest management students consists in identifying, understanding and building a mental model (diagnosis) of the ecosystems specific to various forest parcels in order to evaluate the impact of action decisions, such as cutting down trees (for forest thinning) or setting up public reception facilities etc., on the preservation of these ecosystems. The overarching goal of the present research is to contribute to the construction of such models.

The volume of research into IVR-assisted learning is currently growing apace, see for example the very recent studies by Makransky and Petersen, 2019; Makransky et al., 2020b,c; Stenberdt and Makransky (2023), Albus et al. (2021), Petersen et al. (2022), and Makransky and Mayer (2022) among many other articles and see also below.

Despite the popularity of IVR, which allows a high level of perceived presence, user-control and agency [see the CAMIL by Makransky and Petersen (2021), and below], a number of recent studies have revealed that IVR does not always have positive effects on learning compared to conventional desktop multimedia or video presentations (Parong and Mayer, 2018; Makransky et al., 2019a,b, 2020a; Makransky, 2022; Mayer et al., 2022). As stated in the title of the article by Makransky et al. (2019b), *“Adding immersive virtual reality… causes more presence but less learning.”* In a recent review, Mayer et al. (2022) showed that out of 13 studies comparing IVR with more conventional media (or computer-screen VR) such as desktop, slideshow or video, seven indicated that students learnt better with conventional media than in IVR environments.

Only five studies showed a positive effect of IVR, with most of them revealing a small effect size (Cohen’ *d* from 0.10 to 0.29). In their meta-analysis, Wu et al. (2020) found a small advantage of IVR over more conventional technologies. In their own meta-analysis, Coban et al. (2022), considered fourteen previous meta-analyses and 105 independent ESs (Effect Sizes) from 48 primary studies: They found an overall ES of g = 0.38, which corresponds to a small to medium positive effect. Araiza-Alba et al. (2020a,b) arrived at a similar conclusion concerning the use of desktop VR and IVR with children.

The high degree of realism, the enriched nature of the visual information, the potentially overwhelming effect of an immersive visual field with perceptual saliency effects, the number of elements, the user-control and agency factors may lead not only to attentional distraction and disorientation but also to an increase in both extraneous and germane cognitive load (Sweller et al., 2011; Albus and Seufert, 2022; Mayer et al., 2022). According to Makransky (2022), *“The immersion principle in multimedia learning is that immersive virtual environments promote better learning when they incorporate multimedia design principles. In short, immersive media do not necessarily improve learning but effective instructional methods within immersive virtual environments do improve learning” (page 296)*.

In this way, one recent line of research has consisted in “systematically” testing the potential benefits and effectiveness of making use of multimedia principles and features in IVR learning environments (Albus et al., 2021; Albus and Seufert, 2022; Mayer et al., 2022). Features that have proven their effectiveness in multimedia learning (Mayer and Fiorella, 2022) include, for example, the principles of instructional design and generative activities. The review by Parong (2022), reported a positive significant added value of features such as modality, personalization, pre-training summarizing, answering, enacting, and gender matching on learning performances in 9 out of 12 studies. Recently, Stenberdt and Makransky (2023) tested the feedback principle in IVR-based material promoting pro-environmental waste-sorting behavior.

The present study tested another instructional design feature, namely the effect of visual signaling associated with feedback for teaching agriculture students about forest ecosystems using a realistic living forest simulator in IVR conditions. To the best of our knowledge, there are to date only very few published studies that report results and have investigated verbal signaling in IVR, Albus et al. (2021) and Zhang et al. (2023), while another recent study (Decker and Merkt, 2023) has focused on the effect of signaling cues in desktop VR and IVR environments.

### 1.1 Learning in IVR

A second line of research, conducted within the theoretical framework of the CAMIL (Makransky and Petersen, 2021), has consisted in investigating the effect of the two main immersive affordances, namely presence and agency, on cognitive and affective factors, i.e., enjoyment, interest, self-efficacy, self-regulation, embodiment, cognitive load (extraneous, germane and intrinsic), and the effect of these factors on learning outcomes. In addition, new and interesting analytical methods, such as structural equation models (SEM) and mediation techniques, have been used to investigate whether cognitive and affective factors could mediate a hypothesized effect of IVR on learning performances (immediate and/or delayed retention and comprehension post-tests, as well as transfer post-tests etc.). For example, Makransky and Mayer (2022) investigated the benefits of taking an IVR-based climate-change-related virtual trip by comparing two groups of students (13 to 16 years old), one taking the virtual trip to Greenland via a head mounted display (HMD) and the other using 2D video. The results showed that the HMD group outperformed the 2D video group on presence, enjoyment, interest and retention in both the immediate and delayed post-test. The SEM analysis showed that enjoyment mediated the pathway from instructional design to immediate post-test performances, while interest mediated the pathway from instructional design to delayed post-test performances. Recent studies conducted within the same framework have demonstrated a positive effect of IVR and interactivity on enjoyment, interest, self-efficacy, expected outcomes, perceived embodiment, spatial presence, motivation and behavioral intention changes compared to the same content presented using 2D desktop or conventional multimedia technologies, and have done so in very different fields and subject areas (Ahn et al., 2022; Andersen et al., 2022; Makransky and Klingenberg, 2022; Makransky and Mayer, 2022; Petersen et al., 2022; Vandeweerdt et al., 2022; Plechatà et al., 2022a,b; Bagher et al., 2023; Stenberdt and Makransky, 2023). A similar pattern of results was found with young children asked to perform tasks such as remembering and recalling a story and the related emotions (Araiza-Alba et al., 2020a), understanding and recalling seaside safety instructions (Araiza-Alba et al., 2021b) and problem solving (Araiza-Alba et al., 2021a).

However, while these studies have often shown an effect of IVR on mostly perceived cognitive and affective factors, they have not always consistently demonstrated an effect on learning performance (see also Makransky, 2022), even when moderating factors have also been taken into account. Further, and as expected by the CAMIL, studies have revealed that IVR-based learning results in an increased cognitive load, and especially extraneous and germane cognitive load. This could account for the lack of effect observed on learning performances and outcomes. Cognitive load has been measured using direct brain measures (EEG) and subjective scales (Makransky et al., 2019b; Breves and Stein, 2022). The effect on cognitive load was significant in several studies. For example, Baceviciute et al. (2021) found that it was cognitively more demanding (EEG measures) and less time-efficient to read one and the same text in an IVR environment compared to real physical reading. Furthermore, in studies testing the modality effect, Baceviciute et al. (2020) revealed that reading was superior to listening for the learning outcomes of retention, self-efficacy, and extraneous attention. Reading text from a virtual book was reported to be less cognitively demanding than reading from an overlay interface. EEG analyses showed significantly lower theta and higher alpha activation in the audio condition.

The results of these previous studies led to the first line of research mentioned above, which tested the potential benefit of including multimedia principles in IVR learning environments. So far, only a few studies have been conducted (but their number is growing) and the initial results seem mixed: for some principles, the IVR results were similar to those obtained with multimedia 2D documents, whereas for others, they appear to be different (Mayer et al., 2022). This is, for example, the case for the modality principle, Baceviciute et al. (2020). Recently, Albus and Seufert (2022) also identified a reverse modality effect in VR: learning performances were better in the visual-only than the audio-visual condition as measured on recall, comprehension and transfer, with extraneous cognitive load being similar in the two conditions. Furthermore, Klingenberg et al. (2022) found that compared to a control condition, adding segmentation or summarizing activities to an IVR science lesson resulted in better transfer in seventh grade students but not in more factual knowledge. Combining segmentation and summarization did not improve learning. The body of research on IVR learning also reports many differences across studies on factors such as the type of task and contents (science lessons, architecture, biology, history, science lab environment), declarative or procedural knowledge [see the meta-analysis by Coban et al. (2022)], participants’ age and activities, the task requirements and also the type of technical implementation: HMD IVR, 360° video, 2D on-screen VR, and also the level of rendering of the VR technique used. Such factors could account for at least some of the heterogeneity among the observed results. It is also necessary to address the question of signaling techniques and this issue will be addressed in Section 1.3 below after the theoretical background to IVR learning has been presented.

### 1.2 Theoretical background: “The great forgotten factor,” visual perception?

In visually rich IVR environments displayed in 3D, we might hypothesize that the distribution of priorities between text and pictures changes. In “conventional” multimedia documents, which often include a limited number of pictures that are sometimes “poorly” designed compared to IVR or full HD videos, text is dominant, and previous studies have shown (see, for example, Schmidt-Weigand et al., 2009) that learners spend much more time on the text than on the pictures. The reverse may be true of IVR presentations because the 3D and presence effect emphasize the pictures. The cognitive guidance and strategies used by the learner should therefore rely less exclusively on the text. The feeling of the *visual completeness* of the environment due to the 3D space and the presence feeling that results from effects of perceptual salience and immersion may make the visual channel much more dominant than the phonological channel, meaning that the visual sketchpad would become the priority processing mechanism and representational system, taking precedence over the phonological and word representation system. This hypothetical explanation could partly account for the reverse modality effect observed in the study by Albus and Seufert (2022).

The present study follows on from research assessing the use of multimedia principles relating to visual signaling/cueing in IVR environments conducted within the framework of the CAMIL (Makransky and Petersen, 2021) and the CTML (Mayer and Fiorella, 2022) models.

However, because of the potential dominance of perceptual visual and pictorial processing in IVR, which could impose a visual load (rarely or never measured *per se*, Skulmowski, 2023), another complementary processing model may be of interest: The Animation Processing Model, APM (Lowe and Boucheix, 2008, 2016). IVR environments dominated by pictures frequently involve complex 3D spatial information and also dynamic, e.g., transient, information. On the one hand, the spatial information not only includes depth views, e.g., depth perception, but also front, back, side, top and bottom views of the display. On the other hand, a transience effect may arise from the possible dynamics of the objects, the context and the learner’s body movements in the scene when exploring these rich environments. As postulated by the APM (Lowe and Boucheix, 2008), understanding and building mental models from such complex pictorial environments involve (i) pre-attentive (gestalt theory principles) and (ii) perceptual processes, which are the bases (“raw material”) for the (iii) cognitive processing of concepts, causal relations, knowledge building and memorization. Perceptual processes lead to the required spatial and temporal partitioning of the content elements in the IVR display. The efficiency or relevance of partitioning the scene/content may depend on (i) the achievement of an optimal alignment of the perceptual salience of the (dynamic) pictorial information, (ii) top-down application of the learner’s prior knowledge (iii) the learner’s visuo-spatial abilities. Perceptual partitioning seems to be necessary in order to allow effective cognitive processing when selecting, organizing and structuring the relevant visual information (Mayer and Fiorella, 2022). In line with the foregoing, the APM proposes a five-stage model of the processing of dynamic visuospatial information, involving both the decomposition and composition of the visual information (Lowe and Boucheix, 2008; Boucheix et al., 2013; Lowe and Boucheix, 2016; Lowe et al., 2022).

Stage 1. *Localized perceptual exploration*. After a very short holistic processing, the continuous flow of spatiotemporal information is perceptually parsed into small groups of neighboring graphic entities in order to identify event units. This takes place through both pre-attentive and attentive exploratory processing. Stage 2. *Regional structure formation*. Event units, as well as spatiotemporal entities, begin to link up locally to form regional structures representing various parts of the display. General causal relations begin to form between these regional event units. This processing leads to the formation of dynamic micro-chunks (and micro-chunks of entities), which can be considered as individual *islands of activity and islands of comprehension* that correspond to what is happening in different regions spread across the display area. Stage 3. *Global characterization*. During this phase, the learner develops a more global internal characterization of the dynamics of and relations between spatiotemporal micro-chunks. The islands of activity are linked into broader coherent structures, such as domain-general causal chains. Stage 4. *Functional differentiation*. The relational structure is characterized in a domain-specific way. Actions are propagated along causal chains and/or bigger visual chunks. Events are interpreted in terms of the referent’s central objective and the different subsystems are considered as contributing to the overall functioning of the system. This processing identifies functional episodes. Stage 5. *Mental model consolidation*. Mental models are thought to facilitate the understanding of a system’s behavior, not merely in a single situation but also in a variety of circumstances and across varied operational requirements. Furthermore, the model should make it possible to adapt to different task requirements and performances. This processing results in a flexible mental model.

We assume that several features of the APM could be applicable to other forms of complex and rich visual displays, such as IVR. For example, the decomposition phase, involving the parsing of the relevant spatiotemporal entities of the displays at different locations in the immersive environment (which can be distant from each other), and the composition phase, in which relational systems are created between spatiotemporal entities and levels (macro and micro), may be also involved in IVR learning. How is it possible to help learners process such a complex visual environment?

### 1.3 The potential benefit of signaling in IVR

Because of the dominance of spatiotemporal and pictorial information in IVR, and based on the APM model, adding visual signaling related to task-relevant information in IVR could help learners direct their attention to relevant information, thus enabling them to select, organize, structure and integrate visual information at the right location at the right time (Mayer and Fiorella, 2022; Van Gog, 2022). Such cueing could reduce visual load, assist in the inhibition of irrelevant information and create relations between relevant information (chunks, causal chains, etc.). In the context of recognizing forest flora and fauna, for example, cueing may help learners navigating in the IVR forest environment to select tree, flower or plant species and choose soil types that promote biodiversity and forest preservation.

#### 1.3.1 Signaling in conventional multimedia learning

The signaling principle holds that people learn better when signals/cues (verbal and/or visual) are added, not to give more information but to highlight the relevant information and the organization of the multimedia presentation (text and/or pictures). Verbal signals include, for example, adding pointer words, numbering etc. Visual signals include highlighting, spotlighting, zooming, color coding and graphic organization (Fiorella and Mayer, 2022; Van Gog, 2022). Cueing usually introduces and increases the visual contrast between the signaled information and the other parts of the display. In this way, cueing directs learners’ attention toward the relevant information and cuts search times and the extraneous load related to the search activity.

The results of numerous previous research studies have reported a positive effect of signaling on multimedia learning (for example de Koning et al., 2007, 2009, 2010a,b; Boucheix and Lowe, 2010; de Koning et al., 2011; Boucheix et al., 2013). Three main meta-analyses have reported that signaling improves learning effectiveness, with effect sizes ranging from small to medium being observed. Richter et al. (2016) analyzed the efficiency of signals that highlight correspondences between text and pictures and found a small positive effect size (*r* = 0.17) in favor of signaled multimedia for transfer and comprehension. Schneider et al. (2018) used a larger variety of multimedia material that included standalone dynamic visualizations and revealed a small-to-medium positive effect size of cues on retention (g + = 0.53) and transfer (g + = 0.33). Finally, Alpizar et al.’s (2020) meta-analysis showed a small-to-medium effect size in favor of signaling (*d* = 0.38). However, eye-movement data has also indicated that even if signaling is successful in directing the learner’s attention to the specific relevant information, it does not always guarantee a significant improvement in comprehension and transfer performance (de Koning et al., 2010a; Lowe and Boucheix, 2011). In the case of dynamic presentations (animations), comprehension is improved by signaling techniques that not only direct the learner’s attention but also emphasize the structure of the learning material (de Koning et al., 2009) or the relation between elements, such as causal chains for example (Boucheix and Lowe, 2010). Furthermore, dynamic signaling improves the comprehension of transient information more than static signaling (Boucheix et al., 2013).

#### 1.3.2 Levels of signaling in complex visual IVR

As mentioned above and as far as we are aware, little previous research has investigated the signaling effect in IVR. The study conducted by Albus et al. (2021), used signals in the form of textual annotations (written labels) during learning from a 3D animation on the subject of seawater desalination presented with a low end VR-HMD set-up. The effect of the presence or absence (control group) of signals on learning outcomes and extraneous and germane cognitive load was investigated. Results showed that written annotation signals improved learners’ recall performance and germane load compared to the control group but did not improve either deeper processing (comprehension and transfer) or extraneous cognitive load. Adding textual annotations might therefore be an appropriate approach in IVR learning situations. However, it seems that the written annotation signals used in the learning material of this study were written localized “repetitions” of the aural audio information delivered by the animation, and it is therefore possible that there was a redundancy effect (Fiorella and Mayer, 2022; Kalyuga and Sweller, 2022) that weakened the signaling effect. Whatever the case may be, Albus et al. (2021) confirmed the signaling effect and also suggested that other forms of signaling should be tested, in particular dynamic signals (Boucheix and Lowe, 2010; Boucheix et al., 2013). In their recent investigation, Zhang et al. (2023) used a VR training simulation of the process of assembling computer hardware and confirmed that textual cues boosted immediate knowledge gain (retention) but did not improve transfer.

In the present study, we focused on testing the potential benefits of visual signals rather than relying exclusively on textual signals. We used a typical training task given to agricultural forestry students. In this task, the students have to identify the specific ecosystems and biodiversity of forest parcels before deciding on whether and how to intervene (by cutting trees, for example). Both comprehension and diagnostic activity are required in order to identify the quality of the ecosystems of the parcels. Due to the information density and the potential *“sense of proprioceptive visuospatial comprehensiveness”* in IVR, we propose that not only one but several levels of signaling could be needed in order to help users better organize the relevant information in the environment. Different signaling levels could then highlight different levels of the information structure of the visual environment: in the forest scenario, for example, these could include the level of trees and plant-related information and the level of soil information (including soil moisture levels and wildlife information). This type of differentiated signaling would not only highlight the structure of the relevant information but also could direct attention toward conceptual relations that might link the signaling levels: for example, between the soil composition level and the tree and plant level. However, using too many signals could impose a greater perceptual and cognitive load.

### 1.4 Feedback with generative activities in IVR and signaling

The provision of informative feedback is widely recognized to be an effective instructional method that enhances learning performance and increases the learner’s engagement (see, for example, Hattie and Timperley, 2007; Hattie, 2008). According to the feedback principle, students in multimedia learning situations learn better with informational and explanatory feedback than with only corrective feedback (Johnson and Marraffino, 2022). Recently, and interestingly, Stenberdt and Makransky (2023) used two types of feedback in a study which investigated the effect of mastery experiences in IVR on promoting pro-environmental waste sorting behavior. “Conventional” corrective feedback was compared to an exaggerated feedback which consisted in showing learners the effect of incorrect waste management activities on the environment. The depicted environmental changes were exaggerated so that sorting one waste item (incorrectly) reflected the effect of many people persistently sorting this type of waste in the same way.

In the present study, informative feedback was given to learners during the training task, in particular after they had made a first action decision based on their diagnostic of the forest ecosystem and before making a second action decision. We expected the feedback principle to impact task performance, in particular given that during the learning-by-exploration time in the IVR environment, the learners were actively able to use the joysticks associated with the IVR headset to navigate toward specific forest elements (trees, plants, soils etc.) and, on reaching them, obtain information about their nature and characteristics (see the details in the Method section).

We are not aware of any previous research that has studied the combined effect of signaling and feedback in multimedia-based or IVR learning. We hypothesized that there would be an interaction effect of signaling and feedback on task performance. We speculated that the cognitive processing mechanism underlying such an interaction could act as follows. The effectiveness of the cognitive processing and interpretation of information feedback may depend on the relevance and quality of the knowledge and information acquired and memorized immediately beforehand during the task (also including prior knowledge). If so, the cued items of information preselected for learners in a signaling condition, which are relevant by definition, limited in number and reveal the underlying structure of the information, might be better retrieved from working memory and processed for treating the feedback information than in a non-signaling condition. In this latter condition, the internal activation of more information, which is potentially irrelevant and understructured, would not facilitate the cognitive processing of the feedback information. In other word, the signaling of task-relevant information could make it easier to establish the necessary internal relations between the content of the feedback information and the content of the previous information memorized during the task before this feedback and indeed improve these relations. As a consequence, the positive effect of feedback on task performance might be greater when signaling is used than when it is not.

### 1.5 The present study

As mentioned above, the present study investigated the influence of two levels of visual signaling and information feedback in a vocational training task involving (i) the exploration, and interrogation (forest elements searching), of forest areas for the purpose of diagnosing the ecosystem and (ii) two subsequent decisions, including a justification given by the learner: a first decision before information feedback on the forest elements present in the areas visited during the exploration and a second decision after this feedback. The action decisions made by forestry professionals and students in agricultural colleges in order to ensure forest sustainability, for example whether to cut trees, how to manage plots, etc., must result from the application of knowledge about biological ecosystems and lead to a diagnosis of the biological potential of the forest or the parcel. By learning and applying such knowledge, learners are able to predict the effect, whether positive or negative, of human action decisions on forest development (trees, plants, animals etc.) in the short (1 to 5 years), medium (5 to 10 years) and long term (more than 20–50 years to 100 years). An IVR environment based on authentic agronomic forestry models could be an effective learning and training tool. The IVR environment used in the present study is a part of a wider project called Silva-Numerica (involving several collaborating partners), which has involved the design, construction and implementation of a realistic live forest simulator which is able to simulate forest development (trees, plants, soil, etc.) and, in particular, the effect of human action on future changes. It makes use of computer algorithms derived from agronomic models.

The project started by testing and implementing a screen-based (desktop) simulator for classroom use (college and undergraduate university levels). In the present study, we used a later IVR version (Figure 1) which induces a high level of perceived presence, including realistic 3D features, and provides verbal and pictorial information on visible and invisible elements during the exploration of forest parcels (types of trees and plants, fungi and mushrooms, soil types, animals, moisture content, micro-habitats, etc., see details in the Method section). We also used a typical training task taken from the agro-ecology curriculum. Students were told to explore (and interrogate) several (small) forest areas in order to decide which one would be the most suitable for building a public reception area with (a forest observatory and) a picnic area and keep the impact on the nearby forest ecosystem to a minimum. This task involves (i) a first phase of exploration-based learning of the areas in order to build a mental model of the characteristics and richness of the ecosystem of each area and (ii) making a decision about the most suitable area for the location of the public reception area. Naturally, the participants were required to give a clear justification for their decisions (see the details in the Method section). The cognitive processing required before making the decision involved comprehension-diagnostic activities: the selection and identification of relevant ecosystem elements (types of trees, trees planned for the future, plant species, fungi, soil features, potential wildlife) and the establishment of relations between the identified elements.

Complementary influencing factors were also controlled for the study. First of all, prior knowledge of participants (agricultural education students) was assessed. Secondly, since the participants had to navigate in forest areas and read verbal information about selected forest elements, their spatial abilities [see the recent paper by Hartmann et al. (2023), on spatial abilities and IVR] and verbal working memory spans were measured and controlled. In addition, and based on the CAMIL model (*cf.* Makransky and Petersen, 2021), several technological features, affordances and cognitive-affective factors were assessed using subjective scales based on the assessment tool proposed by Makransky et al. (2017): perceived realism and authenticity, presence and immersion, perceived task facility-difficulty, usability of the environment’s functionalities, specific knowledge requirements, cybersickness, overall enjoyment during the IVR experience (see Method section for details).

### 1.6 Hypotheses

Four main hypotheses were formulated in the light of the foregoing.

*H1*: We expected an effect of visual signaling on the number of elements (trees, plants etc.) explored, e.g., targeted (and then selected) with the joystick prior to decision-making. In the signaled conditions, we expected learners to target and select fewer elements than in the non-signaled conditions. Targeting fewer items would help participants select the relevant information and organize and integrate it before making a decision.

*H2*: We expected a positive effect of visual signaling on correct (and justified) decisions. This would apply to both the first and second decisions. We did not formulate any hypothesis about the question of whether there might be differences between the levels of signaling, i.e., the signaling of trees and plants (which tended to be at or above standing height) and the signaling about the soils and less visible information (which tended to be at ground level). Both signaling levels are important in order to infer the wealth of biodiversity in the explored area.

*H3*: We predicted that the information feedback provided after the first decision would have a positive effect on performance on the second decision. Information feedback about the forest elements relevant for building a mental model of the ecosystem of the visited forest areas would help participants to make the correct decision. Naturally, the provided information did not indicate which area had the richest ecosystem and biodiversity. However, it did provide an informative description of specific, task-relevant, information about the forest elements present in the area (types of trees, plant species, soil types, animals, fungi and mushrooms etc.).

*H4*: We expected to observe an interaction between signaling and feedback on decision performance, with learners in the signaled conditions outperforming those in the non-signaled condition for the second decision after the feedback. The internal matching between the stored task-relevant information before the feedback and the relevant information provided by the feedback would be facilitated in the signaled conditions, thus facilitating the elaboration of a high-quality mental model of the various forest ecosystems. In the non-signaled condition, learners might store both irrelevant and relevant ecosystem-related information before the feedback, making internal processing of the feedback more difficult and potentially producing subsequent interference.

*H5*: Regarding justifications, we expected a higher number of correct justifications of decisions made in the signaled conditions than in the non-signaled condition. Furthermore, signaling was expected to increase the number of justifications based on considerations of ecosystem biology rather than justifications based on human wellbeing and comfort. Finally, regression and covariance analyses were run to explore the relations between subjective measures, and in particular realism, presence and immersion, perceived task facility-difficulty, usability of the environment’s functionalities and decision performances in the four experimental conditions. This led to H6, namely an expected significant covariant relation between presence and performance, which would be consistent with the immersion principle (Makransky, 2022).

## 2 Method

### 2.1 Participants and design

Eighty-nine students from a French agricultural college took part in this study (average age: 16.19 years, 39 girls and 50 boys, respectively 43.82 and 56.18%). Their curriculum was composed of a common core including general subjects (French, Math, English, Life and Earth Sciences) and two important special options: agricultural production technology and development and outdoor space enhancement with a focus on forestry. Four of the college’s classes were invited to take part in this experiment, i.e., two classes per grade: two 10th-grade classes and two 9th-grade classes. The experimental design involved four groups with two between-subjects factors corresponding to the two signaling levels factors (2×2). One group saw no signaling (no-signal group, *n* = 23), i.e., there was no highlighting of the task-relevant trees and plants by means of flashing colors (F-) and no signaling marker stones on the ground (S-, see Materials section for details); two groups saw either the flashing (highlighted) task-relevant trees and plants (*n* = 21) or the signaling marker stones on the ground (*n* = 22) (F+S- or F-S+, respectively); one group saw both signaling levels, i.e., both the flashing trees and plants and the marker stones on the ground (*n* = 23, F+S+). Feedback was the within-subjects factor for each of the four groups. The 89 students were randomly assigned to the four groups and the groups were matched on prior knowledge, spatial orientation ability and grade (see below).

Previous research into the benefits of signaling on learning or training performances has indicated small to medium effect sizes (see above, Section 1.1). We conducted two power analyses based on a medium effect size for a two-way ANOVA analysis, e.g., with two between-subjects factors corresponding to the two levels of signaling and feedback as a within-subjects factor. A compromise power analysis (compute implied α and power, given the β/α ratio, sample size and effect size) performed with G*power 3.1 (Faul et al., 2007) indicated a β/α ratio of 1 and a power (1- β err.prob.) reaching 0.83 with a sample of 89 participants for an effect size *f* of 0.25. A sensitivity power analysis was also performed with G*Power (3.1, Faul et al., 2007) to estimate the minimum effect size that could be detected by a factorial ANOVA with the four groups (*N* = 21 to 23). This analysis indicated that a medium effect size *f* of 0.28 would be detectable with the current sample size. We used Cohen’s conventions defining small (*d* = 0.20, *ηp^2^* = 0.02, *f* = 0.10), medium (*d* = 0.50, *ηp^2^* = 0.13, *f* = 0.25) and large (*d* = 0.80, *ηp^2^* = 0.26, *f* = 0.40) effects (Cohen, 1988).

The experiment included two sessions: (i) a paper-and-pencil-based pre-test session, (ii) a learning (training) IVR session, which was immediately followed by decision 1 (with justification, performance on post-test 1) before feedback was provided and decision 2 made (with justification, performance on post-test 2). Finally, the subjective post-test questionnaires were administered.

**Figure 2:**
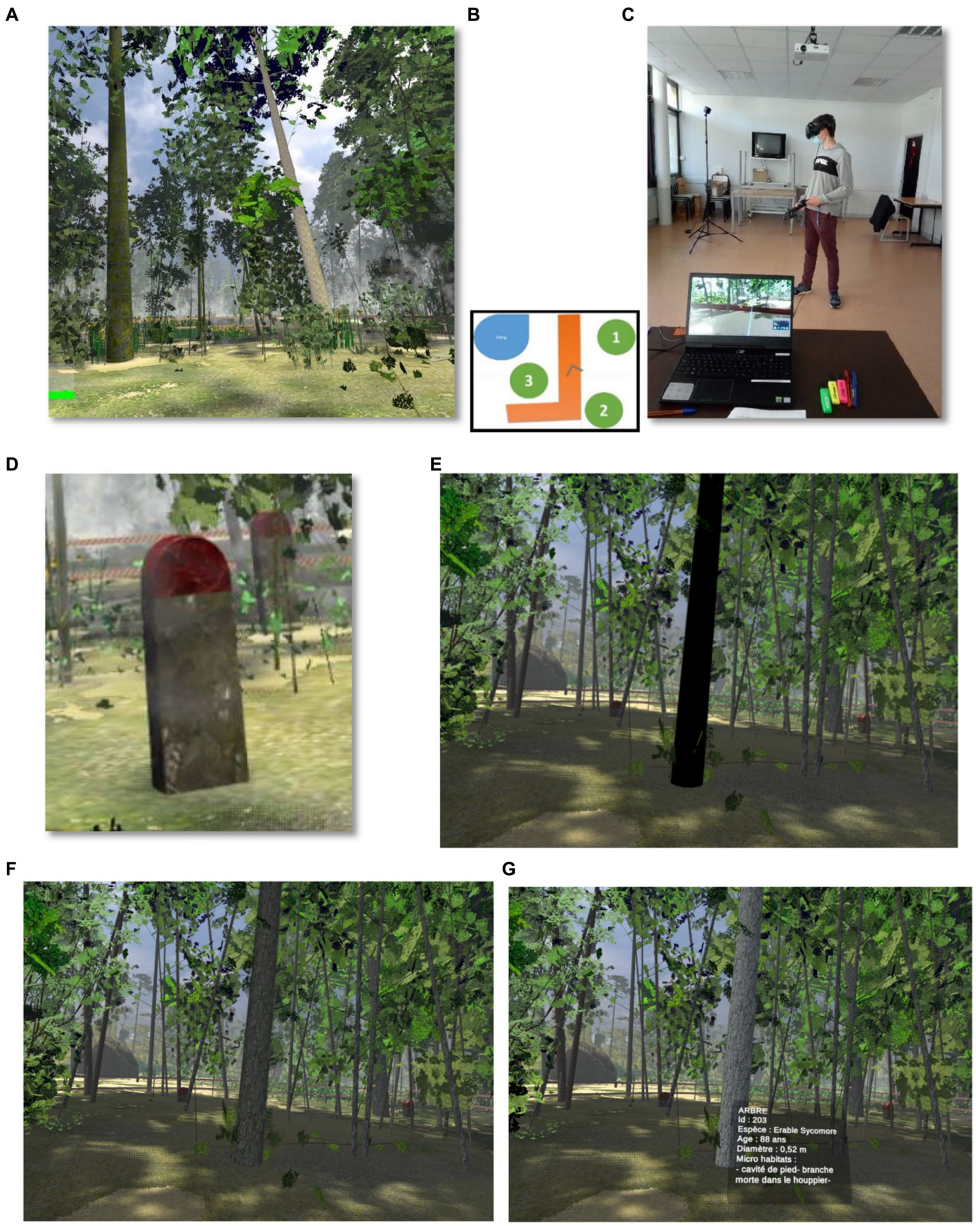
Snapshots of the IVR environment with (A) a forest area; (B) a reproduction of the map accessible in the virtual environment, including a body of water (blue), the three zones (green) and a path (orange); (C) a participant during the task; (D) an example of a marker stone, with the textual information which popped when targeted and selected; (E, F) an example of a flashing tree, and (G) in white, the textual information which popped on a targeted and selected tree: Tree; Id: 203; Species: Sycamore Maple; Diameter: 0.52 meter; Microhabitats: root cavity, dead branch in crown.

The paper-based pre-test session took place in the classroom and the IVR session in a special large room used for virtual reality teaching (see Figure 2). During the pre-test session, students completed a series of tests to assess their working memory and spatial skills and answered a prior knowledge questionnaire on the subject of the IVR exploration: the forest ecosystem. Students’ performance on these tests and their average grade scores in agricultural disciplines, including ecosystems theme, were used to create four homogeneous groups (with *N* = 21 to 23 participants per group) corresponding to the four IVR forest conditions. In the second session, which took place between one and three weeks later, students individually explored and interrogate the forest space in virtual reality using an IVR headset (HTC Vive).

This exercise, involving the learning by exploration of forest areas, was followed by a first decision task. Feedback was then provided, followed by the second decision task. Finally, each participant completed a series of questionnaires: (i) the subjective scales about the IVR experience, (ii) a demographic questionnaire. Each session lasted approximately 60 min and the total duration of the experiment was 120 min. The first session was conducted as a whole class, whereas the participants performed the second session individually. At the end of the first session, students were informed that they would participate in a second immersive virtual reality session, but they were not informed of the nature and content of the task they would have to complete.

### 2.2 Learning material and experimental equipment

#### 2.2.1 Pre-tests

During the pre-test session, participants completed three paper-based tests in order to allow us to form homogeneous controlled experimental groups: (i) a spatial orientation test; (ii) a verbal working memory test and (iii) a prior knowledge test. As it can sometimes be difficult to orient oneself in space when immersed in a VR environment, (see Drai-Zerbib et al., 2022; Mayer et al., 2022), we also asked the participants to perform a spatial orientation test. The spatial orientation test was a French translation of the “Perspective Taking/Spatial Orientation Test” developed by Hegarty et al. (2008). This test was composed of 12 items organized as follows: various objects, such as a car, a house, a cat or a flower, were depicted on a half-page. An instruction displayed below the images asked the participants to imagine themselves in the place of one of the pictures, look at another picture, and from there determine the location of a third item. To answer, they had to draw a line in a circular dial located under the instruction and were given 5 min to complete the twelve items. The Danneman and Carpenter (1980), adapted into French by Desmette et al. (1995), was used to assess the verbal working memory span. In this test, participants are asked to listen to a series of orally presented sentences. At the end of each set of sentences, they must recall the last word of each sentence in the set. Each set consists of 2, 3, 4, 5, or 6 sentences. The participant must therefore listen to the sentences and keep the last word of each sentence active in working memory. The participants in our study had to listen to the series of sentences and then write down the last word of each sentence in the answer booklet, but only after receiving the “go” signal. Two scores were calculated from the students’ responses: the more demanding criterion for scoring the responses was the number of completely successful sets of sentences (without errors or missing words, out of 15), and the less demanding criterion was the number of words correctly and completely recalled in the correct order in all sets of sentences (max. 40). Both scores were transformed into percentages and we used the average of the two scores for data analyses.

The factual knowledge pre-test consisted of two open-ended questions: (i) Define as precisely as possible what a forest ecosystem is, what its characteristics are; (ii) With reference to the ecosystems present, what is the best way to manage a forest area?

We chose this question format, firstly because it uses the types of questions usually asked by teachers to students, and also in order to avoid influencing the answers given during the following experimental phase. Indeed, proposing MCQ encompassing biodiversity, soil characteristics and management of the forest environment could have led the students to reason differently and give different answers during the IVR exploration. In addition, this type of production and generative task is thought to have more discriminant power than an MCQ-type questionnaire (Mayer and Fiorella, 2022).

To rate these written accounts, we developed a coding criteria matrix based on the answers given by a specialist, the life science student’s teacher, to these questions. We used the 10 main criteria (together with their definitions) provided by the teacher: diversity, human intervention, adaptation of flora and fauna (appropriateness), dynamic system, biotope, interaction between species, renewal cycle, stable environment, sustainability of the environment. Each criterion was scored out of 1 as follows: 1 point was awarded to the student when the criterion was present and defined completely correctly; 0.5 points were awarded when the criterion was mentioned by the student in an undeveloped or imprecise way and 0 points were awarded when the criterion was absent or wrongly defined and explained. The experimenter coding of these written data was double checked by the specialist teacher. The scores out of 10 were transformed into percentages for statistical analysis. The prior knowledge pre-test was followed by a demographic information questionnaire that included questions about the participants’ age, gender, use of computers and digital tools such as social networking and video games. We also asked them if they wore glasses and if they knew of any learning disabilities.

#### 2.2.2 Learning material

To permit the use of the HTC Vive HMD IVR headset, we delimited a nine square meter exploration area using sensors installed on tripods at a height of 2 meters. These two sensors were arranged diagonally across the room, each marking the corner of a 3-meter square. This surface was set up and calibrated using the controllers on a Windows 10 “gaming” computer running SteamVR software. This action area allowed users to move safely through an obstacle-free zone. To indicate the boundary of the exploration area, a blue grid appeared in the headset as the user approached it. In this way, users could reorient themselves to stay safely within the boundary while they were exploring and analyzing the forest areas. The environment proposed a simulation-reproduction of typical forest plots of varying ages that can be found in real forest environments in France (see Figures 1, 2). The numerical model and algorithms of the forest environment were based on an agronomic and biological model of forest development built in cooperation with scientific experts and a research laboratory specializing in image computing and AI. The VR implementation was made with Unity.

The aim was to immerse learners in this forest environment so that they could explore and analyze various forest areas featuring the different elements present (different types of trees, herbaceous plants, microhabitats, types of soils with various humidity levels, see below) in order to make a diagnosis of the forest ecosystem spaces and authorize or prohibit the installation of structures intended to welcome walkers and visitors, while having only minimal impact on the ecosystems of the visited forest areas. The students arrived at their diagnosis of the ecosystem by exploring and analyzing three forest areas while also extracting – i.e., targeting and selecting – and linking relevant elements. This enabled them to decide in favor of or against the installation of a public reception area, such as a forest observatory with a picnic area. This task met our needs in terms of research on the cognitive processes related to learning in immersive virtual reality environments and also dovetailed with the pedagogical needs of the learners in their field practice related to their future professional activity.

The students were welcomed individually by one of two experimenters, each accommodated in a room dedicated to virtual reality experimentation in the college. The two rooms were each equipped with a computer running the experimental software and an HTC Vive HMD.

In order to familiarize each participant with the forest environment and the ways in which they could interact with this environment using the controllers, a tutorial including a simple task in some very simple natural areas was administered prior to the main task.

*The tutorial* was composed of three areas delimited by barriers (boundaries). Each of the areas had a marker stones and an herbaceous plant. The goal was to allow the students to become familiar with the IVR environment and with the control options made possible by the joysticks, such as targeting and selecting an element or moving from one area to another, without immediately immersing them in the forest environment.

*The main virtual environment:* This consisted of three areas or zones within the virtual forest. The student’s task was to learn by exploring and analyzing these three zones. While doing so, they consulted (targeting-selecting) the different elements present in each zone (trees, shrubs, herbaceous plants, fungi, soils) and learned their specific characteristics. The students could walk in the area and move around by teleporting into the environment using the joysticks. They could also consult and select items and also cancel these selections.

Each element consulted, targeted and selected was automatically recorded by the computer. A variety of tree species with a wide range of ages and heights was present in each of the three areas: oak, ash, spruce, beech, maple, elm, hornbeam, alder. Regarding the layout of the different zones, the number and types of trees, their ages and heights, as well as the types of shrubs, herbaceous plants, microhabitats, soils, were strictly controlled, naturally in the light of the level of biodiversity of the ecosystem of each of the three areas. The visual density of the trees and plants was also balanced across the three areas. In Zone 1, a wetland was located near a water body and was partly composed of highly biodiverse elements specific to marshy soils, such as iris, ferns, beech and elm trees. A second area, Zone 2, was located away from the water body and had stable soil with moderate biodiversity: mostly oak but also hawthorn, spurge, beech, maple. The third zone was located between the other two zones and had stable soil and rich biodiversity: hawthorn, spurge, maple, ash, hornbeam, beech, oak and spruce. Microhabitats could be found in all three zones and were composed of polypore fungi and mushrooms, woodpecker cavities, dead trunks on the ground etc. Thus, the level of biodiversity was high in each of the zones, preventing the task from being too simple and instead requiring a fine-grained analysis of the area of vegetation (trees, plants etc.) of wood.

This controlled diversity and fine-grained difference in terms of biodiversity (scarcity of plant species, competition between trees, and number of “interesting” trees, e.g.: straight, balanced, vigorous and of high productive value of wood) and soil stability between the three areas required an in-depth analysis of the elements present in each area in order to come to a reasoned decision after the environment had been fully explored. At the end of the period of IVR exploration, each participant had to decide which of the three zones could be used to build a public reception area that made the least possible impact on the ecosystem and biodiversity of the forest (see below).

Each zone was delimited by barriers, and only the elements located inside these three zones were available for consultation. Each zone was composed of approximately 30 trees, 15 shrubs and 20 herbaceous plants present in similar densities (including a visual impression of similar density) in order not to influence the students’ responses. Each zone was generated by a software program reproducing the real forest balance specific to the type of zone created. At any point during the exploration of the forest areas, the students were able to access a map via the trigger on the controller in order to find their way around the virtual environment (Figure 2B). An arrow indicated the student’s position and orientation in real time. Each area had a number on it, allowing the students to find their way around and then subsequently communicate their choices. Three types of numbering were programmed and counterbalanced, as was the order of exploration of the three zones.

Each of the forest elements could be targeted, viewed and selected with the joysticks. Once the element was targeted, a tooltip gave the following written information: the type of element (for example, tree/shrub/herbaceous plant etc.) and its name. For the trees, the information provided consisted of the tree species (type of tree, e.g., oak, beech, alder etc.), its age, diameter, and the presence of dead branches in the crown, microhabitats, woodpecker cavities, polypore fungi and mushrooms or foot cavities (Figure 2G).

In the signaling conditions, whether F+S+, or F+S- F-S+, elements relevant for ecosystem diagnosis were signaled. As mentioned above, two signaling levels were created using a 2×2 design with four groups. The first level was that of trees, shrubs and plants. The signals on the relevant elements consisted of contrasting flashing colors, F. In flashing mode (Figures 2E,F), the elements flashed one after the other, getting darker and then lighter until the student targeted and looked at them. The flashing signals did not prevent participants from identifying the element, a tree for example, from its visual appearance (such as its texture and color). Once the student had looked at an element, it stopped flashing and the student had to look for the next flashing element.

The second signaling level was that of soil information as well as of less visible or invisible information (such as soil moisture, for example). The signals took the form of marker stones S, which were placed at specific locations in each zone (Figure 2D). Once targeted, the marker stones provided written information about the environment that was not directly visible, such as: stable soil, wet soil, presence of frogs, dragonflies, and garter snakes. However, in the non-signaled control condition, F-S-, the same information was accessible and could be obtained by targeting the relevant forest element with the joystick. The verbal justifications given by the students both during and after their periods of exploration were recorded with a voice recorder during the entire experimental phase: (i) during the learning-by-exploration phase and (ii) when explaining the two decisions, before and after the feedback. These verbal justifications were transcribed and the arguments given for each targeted and selected element were listed for each participant. We recorded the total number of elements selected by each student during their exploration and for the two decisions, i.e., before and after the feedback.

#### 2.2.3 Feedback materials

**Figure 3:**
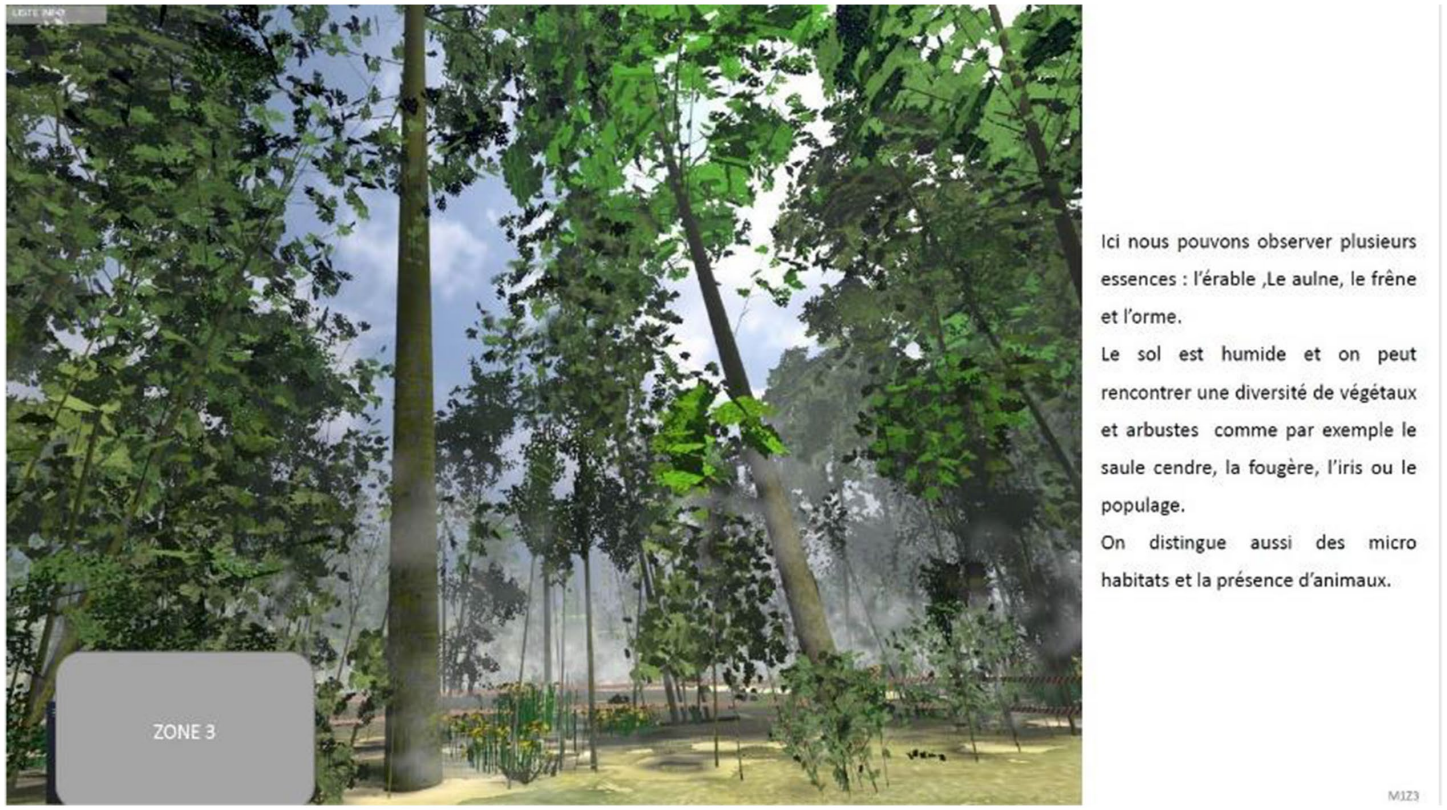
Example of multimedia feedback given to the students. Here is the English version of the textual information on the right of the picture: “Here, we can observe several species: maple, alder, ash and elm. The soil is moist, and a variety of plants can be encountered, including ash willow, fern, iris and marigold. There are also micro-habitats and the presence of animals”.

The feedback (Figure 3) consisted of three A4 multimedia documents, with one page per forest area. Each page consisted of a screenshot of a characteristic frame of the relevant area and a written summary description (approximately 50 words, see Figure 3) of the area’s most important characteristics (types of trees and plants, soil properties – for example soil moisture and soil stability – presence of micro-habitats and animals). This feedback was presented after the learners had made their initial decision in order to allow them to confirm or change it. The information text presented together with the picture was intended to allow them to compare the information stored in memory during the learning-by-exploration phase with the information presented in the feedback and establish internal relations between them. We thought that these relational and comparative activities might make learners aware of the presence of relevant forest elements that they might not have noticed during the exploration phase. They could thus compare the different areas with each other and interpret the role of potential new elements in the light of those found during the IVR exploration.

#### 2.2.4 Subjective scales

Finally, we administered post-test subjective scales presented in a questionnaire about the IVR experience. This consisted of 10 scales (see Appendix A) intended to assess ten items inspired by the CAMIL model and based on the multimodal virtual scale for virtual reality published by Makransky et al. (2017), and in particular on two of the three subscales of the overall questionnaire: physical presence and self-presence. However, although we kept the specific themes from the Makransky et al. (2017) scale, we did not always use the exact wording of the scale, as we were obliged to adapt the items to the particular characteristics of the forest environment and to the nature of the training task we had designed. The 10 items belonged to one of three categories: C1, Presence, including 4 items (immersion, realism, ease of recognition of forest VR elements, ease of graphical recognition); C2: Cognitive Complexity, Extrinsic, including 2 items: ease of movement in the environment, ease of use of interaction features (joystick, map, elements targeting and selection); C3: Cognitive Complexity, Intrinsic, including 2 items: cognitive complexity of exploration of the environment (navigation), cognitive difficulties in the use of specific knowledge about forest elements. In addition, there were two categories with one item each: C4: feeling of cyber sickness; C5: overall enjoyment. For each item, participants had to select a number on a scale from 1 to 5. The questionnaire ended with an open question: “Do you have any comments on the use of the program and virtual reality: strengths/weaknesses, other.” For each of the categories C1, C2 and C3, we used the mean of the items of the category (out of 5).

### 2.3 Procedure

In total, the experiment took 120 min. The first session lasted 60 min. Participants completed the three pre-tests: the verbal working memory test, the spatial orientation test, and the prior knowledge test. They were also asked to complete a demographic questionnaire that included questions about their age, gender, use of computers and digital tools such as social networking and video games, and finally signed a consent form. They were also asked if they wore glasses and if they knew whether they had any learning disabilities. The tests were conducted as a whole class (with N: between 20 and 25 per class) using specific individual booklets and were supervised by three experimenters and a teacher. At the end of the session, the students were informed that they would be invited to a second session in which they would have to perform various activities. The second session took place one to three weeks after the first and also lasted 60 min. The students were received individually by one of the two experimenters. The sessions conducted by the two experimenters took place in parallel in two large rooms of the agricultural college. The experimenter explained the general course of the experiment to the student and then equipped her/him with the virtual reality headset (HTC Vive). The session started with a tutorial in which three uncluttered areas were delimited by barriers. Each of the areas had a shrub and a marker stones. Students were shown the joystick controls and then asked to look at the map, teleport in, look at an item, target and select it. When the learner was confident about using the equipment, he or she was asked to teleport to a specific area of the tutorial. This step took about 10 min depending on how comfortable the participant was with the equipment. The students were then given the task instructions: namely to explore and analyze the three zones and then, at the end of this exploration, to decide in which of them they would choose to set up a public reception facility including an equipped picnic area while respecting the forest ecosystem as much as possible. The students’ task was to explore and visually analyze each area and to target and select the elements that seemed important to the task requirements. Each time they selected an element, they had to justify it. The students had 6 min to explore each zone, i.e., a total of 18 min. When the 6 min were over, the experimenter told them which zone to go to next (counterbalanced order).

After exploring each area, the students removed the virtual reality headset and were asked to say where, in which of the three areas, they would locate the public reception and picnic area and to verbally justify the decision. They then sat at a table where feedback was presented. The experimenter provided the series of multimedia documents, i.e., images accompanied by a short explanatory text. They could then study the feedback images/text document (Figure 3) before making their second decision about the best area, allowing them to confirm their initial decision or change their minds. In both cases, they had to verbally justify the second decision. Finally, participants were asked to complete the subjective scales about their experience with the IVR. The experimenter remained available to answer students’ questions at the end of the experiment and told them not to tell their classmates anything about the content or course of the experiment.

### 2.4 Criteria for coding decision answers

The data for the two decisions was coded by the specialist teacher (of the students’ classes) and the two experimenters.

#### 2.4.1 Criteria for decision coding

We assigned a score between 0 and 1 to each of the two decisions based on the following criteria: 1 point was awarded when the choice of the zone and the corresponding justifications were correct; 0.5 point were awarded when the choice was correct and the associated justifications were partially correct; 0 point when the area chosen was not the correct one or when the area chosen was the correct one but the justifications produced by the student were wrong or absent.

#### 2.4.2 Criteria for coding verbal justification data

To analyze the justification data, we recorded (i) the total number of arguments given by students during the exploration of the virtual forest, and (ii) the number of arguments given during their first and second decisions. For each argument, we coded (i) the correctness of the argument and (ii) the nature of the argument. For correctness, we determined whether the argument was true, false, partial or absent. We assigned a point to the corresponding category (or categories), i.e., true, false, partial, no argument. An argument was considered true when the explanation corresponding to the item was correct, and false in the opposite case. An argument was considered partial when the student provided an incomplete argument. An absence of argument was noted when the student could not justify his or her choice when selecting the element or when making the decision, e.g., “*I know it’s important, but I cannot explain why*.” For the nature of the arguments, we noted the number of arguments given in favor of preserving the ecosystem, − “eco-system protection” argument- and the number of arguments given in favor of human well-being in the environment, − “human welfare” argument. Human welfare arguments may conflict with biological arguments in the field of ecosystem preservation. We distinguished between the three different justification times: area exploration time, first decision time (decision given immediately after exploration), and second decision time (decision given after feedback). The coding of these written data was checked by the specialist teacher.

#### 2.4.3 Coding example for correctness and nature of arguments

*“There are bacteria and fungi and mushrooms so it’s not especially good for the human body, it’s not very hygienic.”* Here, the argument was rated as false and also in favor of human welfare rather than biodiversity in the forest ecosystem.

*“I do not know if it’s beneficial or not, but there are microhabitats, so if after the presence of humans and all that, it takes everything away, it’s important to preserve it.”* This argument was rated as partial, in favor of protecting the forest ecosystem.

*“Holly is not useful so we are taking it out.”* This argument was rated as false (holly is a protected species).

Stable soil marker: *“Well, the soil structure is good. There is no risk of soil collapse or settling.”* Here the argument was rated as true.

#### 2.4.4 Statistical analysis of data

Factorial, mixed ANOVAs and analyses of covariance ANCOVAs were performed to analyze the data. Regression analyses were also conducted in order to analyze the effect of the controlled factors and subjective scales on decision performance. Because the dependent variable “decision accuracy of choices 1 and 2” (including decision answer + justification) varied from 0 to 1 for each decision time, we additionally verified the main results of the ANOVAs by conducting nonparametric, rank-order, Kruskal-Wallis ANOVAs. For all analyses presented in this study, we used *p* < 0.05 as the criterion for significance. Partial eta squared (*ηp^2^*) and Cohen’s *d* values are provided as effect size measures for all main effects, interactions, and *post hoc* comparisons. The *ηp^2^* values of 0.01, 0.06, and 0.14 represent small, moderate, and large effect sizes, respectively (Clark-Carter, 2004), and the Cohen’s *d* values of 0.20, 0.50, 0.80, and 1.3 represent small, moderate, large, and very large effect sizes, respectively.

## 3 Results

### 3.1 Controlled factors

Controlled factors help prevent potential biases when constituting reliable experimental samples. The results for the four controlled factors, prior knowledge, average school grade, verbal working memory span and spatial orientation abilities, are presented in Table 1 for each experimental condition.

**Table 1:** Means (and SD) for each contolled factor (prior konwledge, school grade, working memory, spatial oroientation abilities) in the four experimental signaling conditions, F+S+, F+S-, F-S+, F-S-.

Signaling conditions Participants number: *N*	F+S+	F-S+	F+S-	F-S-
	*N* = 23	*N* = 22	*N* = 21	*N* = 23
Prior knowledge score /10	1.85 (1.02)	1.84 (0.86)	1.50 (0.50)	1.52 (0.68)
School grade /20	11.86 (1.84)	12.03 (1.50)	12.26 (1.45)	12.68 (1.71)
Daneman & Carpenter WM test Correctly recalled words / 40	34.43 (3.35)	33.30 (3.62)	34.37 (4.20)	33.64 (3.38)
Hegarty’s spatial orientation test /12	6.42 (3.32)	6.99 (2.24)	6.46 (3.55)	6.63 (2.45)

No significant effect, main effect or between-groups comparisons were found for any of the four measures in the four ANOVAs conducted, respectively; (i) for the prior knowledge score, *F*(3,85) = 1.29, *p* = 0.28, *ηp^2^* = 0.04, and when the two most different groups were compared (e.g., F+S+ vs. F+S-, Table 1), *F*(1,85) = 2.08, *p* = 0.15, *d* = 0.43; (ii) for the average school grade, *F*(3,85) = 1.10, *p* = 0.35, *ηp^2^* = 0.04, and when the two most different groups were compared (e.g., F+S+ vs. F-S-, Table 1), *F*(1,85) = 2.92, *p* = 0.10, *d* = 0.46; (iii) for the verbal working memory test score, *F*(3,85) = 0.51, *p* = 0.67, *ηp^2^* = 0.017, and when the two most different groups were compared (e.g., F+S+ vs. F-S+, Table 1) *F*(1,85) = 1.06, *p* = 0.30, *d* = 0.32; (iv) for the spatial orientation test *F*(3,85) = 0.17, *p* = 0.91, *ηp^2^* = 0.006, and when the two most different groups were compared (e.g., F-S+ vs. F+S+, Table 1) *F*(1,85) = 0.43, *p* = 0.51, *d* = 0.20. The four groups were homogeneous on the four controlled factors.

### 3.2 Information selection during exploration of the environment and effect of signaling on decision performances

**Figure 4:**
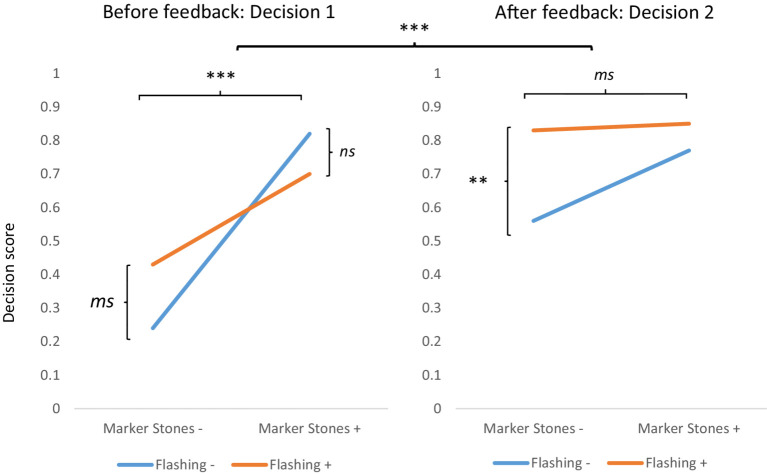
Mean decision scores in each signaling condition: for decision 1 before the feedback and decision 2 after the feedback, respectively. Statistical *p* values: ***p < 0.0001; **p < 0.01, *ms* = marginally significant, *p* < 0.10, *ns*, no significant.

Results are presented in Table 2 and Figure 4.

**Table 2:** Means and SD for, respectively, the number of selected elements and the decision performances before and after the feedback in the four experimental signaling conditions, F+S+, F+S-, F-S+, F-S-.

Signaling conditions Participants number: *N*	F+S+	F-S+	F+S-	F-S-
	*N* = 23	*N* = 22	*N* = 21	*N* = 23
Mean number of *selected (targeted) forest elements* during the exploration phase (sum for the three areas)	50.89 (29.25)	85.51 (60.73)	35.98 (13.87)	83.37 (70.42)
Decision ratio (correct justification): *decision 1, Before feedback*	0.70 (0.33)	0.82 (0.29)	0.43 (0.45)	0.24 (0.33)
Decision ratio (correct justification): *decision 2, After feedback*	0.87 (0.22)	0.77 (0.30)	0.83 (0.33)	0.56 (0.38)

Regarding the number of selected elements, the factorial ANOVA with flashing and marker stones as two between-factors showed that the number of selected elements was significantly lower when first-level signals, i.e., flashing, were present *F*(1,85) = 13.09, *p* < 0.001, *ηp^2^* = 0.15. As expected, this result reveals that this signaling level directly affects the attention learners pay to relevant information. However, with regard to the second level of signaling, the effect of the presence of marker stones was not significant, *F*(1,85) = 0.57, *p* = 0.45, *ηp^2^* = 0.007.

With regard to decision performances, a mixed ANOVA with flashing and marker stones as two between-factors, and feedback, measured on the basis of decision 1 before feedback and decision 2 after feedback, as dependent repeated measures indicated a significant positive main effect of the marker stones signaling level *F*(1,85) = 18.91, *p* < 0.0001, *ηp^2^* = 0.18; a marginal main effect of the flashing signaling level, which failed to reach significance, *F*(1,85) = 2.97, *p* = 0.088 *ηp^2^* = 0.03; and a strong main effect of feedback, *F*(1,85) = 41.64, *p* < 0.00001, *ηp^2^* = 0.33. The interaction between flashing and marker stones revealed a marginal effect, *F*(1,85) = 3.72, *p* = 0.057, *ηp^2^* = 0.042 (see Figure 4 for a better visual representation of the interactions). This interaction showed that in the absence of marker stones, tree/plant flashing improved decision performance, whereas it induced no change in performance when marker stones are present.

Furthermore, the interaction between feedback and flashing was significant, *F*(1,85) = 5.01, *p* = 0.027, *ηp^2^* = 0.055, as was the interaction between feedback and marker stones, *F*(1,85) = 20.46, *p* < 0.0001, *ηp^2^* = 0.19. While a positive effect of the presence of the tree/plant flashing signaling level was observed on decision 1, this positive effect increased significantly after the feedback for decision 2. In addition, the detrimental effect of the absence of marker stones level-2 signaling was largely compensated for by the feedback.

Two factorial ANOVAs were conducted to explore the performances on each decision. One ANOVA was performed for each decision, with the two levels of signaling, i.e., tree/plant flashing and marker stones, as the two between-factors and decision 1 or 2 as the dependent measure.

For decision 1, a main effect of the presence of marker stones was found, *F*(1,85) = 31.53, *p* < 0.00001, *ηp^2^* = 0.27, whereas there was no effect of the presence of flashing, *F*(1,85) = 0.20, *p* = 0.65, *ηp^2^* = 0.002 but a significant interaction between flashing and marker stones, *F*(1,85) = 4.29, *p* = 0.04, *ηp^2^* = 0.05. The analysis of this interaction revealed a marginal significant difference in favor of the presence of flashing when there were no marker stones, *F*(1,85) = 3.12, *p* = 0.08, *d* = 0.47; but when marker stones were present, no difference was observed whether flashing was present or not, *F*(1,85) = 1.34, *p* = 0.25, *d* = 0.39.

For decision 2, the ANOVA revealed a main effect of flashing *F*(1,85) = 7.58, *p* = 0.007, *ηp^2^* = 0.08, a marginal effect of the presence of marker stones *F*(1,85) = 3.38, *p* = 0.07, *ηp^2^* = 0.04, and no interaction between the two factors *F*(1,85) = 1.67, *p* = 0.20, *ηp^2^* = 0.02.

Finally, in order to extend and improve the reliability of these analyses, which were conducted with dependent measures including a three-point scale (e.g., 0, 0.5, 1) for each decision (e.g., 5 point scales for the two decisions), we subjected each decision to a between-groups Kruskal-Wallis ANOVA, non-parametric by rank. These analyses confirmed the previous results. For decision 1, significant differences between the four groups were found, *H* (3, *N* = 89) = 26.03, *p* < 0.00001, with the following mean Rank order: F-S-, R = 27.26; F+S-, R = 38.47; F+S+, R = 53.48; F-S+, R = 60.90. For decision 2, significant differences between the four groups were also found, *H* (3, *N* = 89) = 11.27 *p* = 0.01, with the following mean Rank order: F-S-, R = 32.28; F-S+, R = 45.13; F+S-, R = 51.19; F+S+, R = 51.93.

Taken together, these results for decision performances after the learning-by-exploring phase suggest a significant trend toward a cumulative positive, bounded effect (see Table 1) of the two signaling levels. This point will be further discussed in the final section.

### 3.3 Analysis of justifications

The results for justifications are presented in Table 3. The two types of justification criteria (respectively, quality: true, partial, false and absent, and theme of the argument: biodiversity and human well-being) were analyzed for exploration time, decision 1 and decision 2. Factorial ANOVAs were performed on the number of true arguments and on the theme of the argument -biodiversity- in each learning phase.

**Table 3:** Mean number of arguments (and SD) provided in each phase of the task (exploration time, decision 1 before feedback, decision 2 after feedback) and for each type of argument (true, partial, false, absent and biodiversity or human), in the four experimental signaling conditions, F+S+, F+S-, F-S+, F-S-.

Task stage	Condition Argument type	F+S+ *N* = 23	F-S+ *N* = 22	F+S- *N* = 21	F-S- *N* = 23
Exploration time	True	**4.56 (3.90)**	**3.05 (2.33)**	**3.38 (3.23)**	**3.04 (3.75)**
	Partial	9.83 (4.61)	8.71 (5.26)	8.90 (4.50)	9.35 (4.44)
	False	2.13 (2.07)	1.62 (1.46)	0.71 (1.05)	1.43 (1.62)
	Absent	5.52 (4.50)	4.19 (4.39)	6.47 (6.64)	5.60 (4.81)
	Biodiversity	**7.39 (4.45)**	**4.27 (3.25)**	**6.52 (6.62)**	**3.96 (3.28)**
	Human	2.26 (3.27)	2.27 (2.43)	0.81 (1.56)	0.82 (1.46)
Decision 1 before feedback	True	**1.52 (1.08)**	**1.31 (1.13)**	**0.52 (0.75)**	**0.56 (0.78)**
	Partial	1.65 (1.19)	1.04 (0.78)	1.86 (0.85)	1.48 (1.12)
	False	0.17 (0.49)	0.18 (0.66)	0.28 (0.47)	0.52 (0.67)
	Absent	0.35 (0.65)	0.27 (0.70)	0.48 (0.81)	0.30 (0.47)
	Biodiversity	**1.09 (0.99)**	**0.77 (0.92)**	**0.76 (0.83)**	**0.48 (0.73)**
	Human	0.04 (0.20)	0.14 (0.35)	0.38 (0.59)	0.26 (0.62)
Decision 2 after feedback	True	**1.09 (1.24)**	**0.54 (0.86)**	**1.67 (1.24)**	**0.91 (1.08)**
	Partial	0.69 (0.93)	0.23 (0.53)	1.09 (0.99)	1.08 (1.04)
	False	0.01 (0.01)	0.09 (0.29)	0.14 (0.48)	0.35 (0.77)
	Absent	0.001 (0.001)	0.09 (0.30)	0.28 (0.64)	0.08 (0.42)
	Biodiversity	**0.74 (0.75)**	**0.41 (0.85)**	**1.14 (1.15)**	**0.69 (0.76)**
	Human	0.08 (0.29)	0.04 (0.21)	0.28 (0.29)	0.13 (0.34)

For the exploration phase, no effects were found on the number of true arguments, i.e., no main effect of flashing, *F*(1,85) = 1.52, *p* = 0.22, *ηp^2^* = 0.017 or marker stones *F*(1,85) = 0.79, *p* = 0.37, *ηp^2^* = 0.009, and no interaction between the two factors, *F*(1,85) = 0.58, *p* = 0.44, *ηp^2^* = 0.006. However, for the theme of the argument, we found a main effect of flashing on the number of biodiversity arguments, *F*(1,85) = 8.61, *p* = 0.0004, *ηp^2^* = 0.09, but no effect of the marker stones signals, *F*(1,85) = 0.37, *p* = 0.54*, ηp^2^* = 0.004 and no interaction, *F*(1,85) = 0.08, *p* = 0.78, *ηp^2^* = 0.0009. In addition, there was a difference between the most signaled condition (F+S+) and the non-signaled condition (F-S-): *F*(1,85) = 6.50, *p* = 0.012, Cohen’s *d* = 2.01.

For decision 1, the factorial ANOVA revealed a main effect of marker stones on the number of true arguments, *F*(1,85) = 17.82, *p* < 0.0001, *ηp^2^* = 0.17, but no effect of flashing, *F*(1,85) = 0.11, *p* = 0.77, *ηp^2^ =* 0.001, and no interaction [*F*(1,85) = 0.44, *p* = 0.51, *ηp^2^* = 0.005]. For the theme of the argument, the number of biodiversity-based justifications was higher in the signaled conditions, i.e., for both flashing and marker stones (F+S+ and F-S+), but the differences failed to reach significance, respectively for flashing, *F*(1,85) = 2.57, *p* = 0.11, *ηp^2^* = 0.03, and for marker stones *F*(1,85) = 2.78, *p* = 0.09, *ηp^2^* = 0.03, and the interaction was also not significant, *F*(1,85) = 0.0006, *p* = 0.93. However, there was a difference between the most signaled condition (F+S+) and the non-signaled condition (F-S-): *F*(1,85) = 5.55, *p* = 0.021, Cohen’s *d* = 0.84.

For decision 2, the factorial ANOVA showed, on the one hand, that flashing led to a significant increase in the number of true arguments after the feedback, *F*(1,85) = 7.48, *p* = 0.007, *ηp^2^* = 0.08, and, on the other, that learners who did not see a marker stones during the exploration phase produced significantly more true arguments after feedback *F*(1,85) = 4.005, *p* = 0.48, *ηp^2^* = 0.045. This is consistent with the strong significant effect of feedback on the elaboration of true arguments for decision 2. There was no interaction between flashing and marker stones [*F*(1,85) = 0.20, *p* = 0.65, *ηp^2^* = 0.002]. Similar results were found for the theme of the argument. On one hand, flashing led to a significant increase in the number of true arguments after the feedback, *F*(1,85) = 4.24, *p* = 0.042, *ηp^2^* = 0.05, while, on the other, learners who had not seen a marker stones during the exploration phase produced marginally more true arguments *F*(1,85) = 3.35, *p* = 0.07, *ηp^2^* = 0.037. There was no interaction between flashing and marker stones [*F*(1,85) = 0.10, *p* = 0.76, *ηp^2^* = 0.001].

### 3.4 Complementary subjective scales and performances

Results for the 5 categories of items (Presence, Cognitive Complexity -Extrinsic-, Cognitive Complexity – Intrinsic-, Cyber-sickness and Enjoyment) are presented in Table 4 (see Appendix A for the exact wording of the items).

**Table 4:** Means (and SD) for each of the subjective scale categories and items (out of 5 points) for presence, cognitive complexity-extrinsic, cognitive complexity-intrinsic, sickness and enjoyment in the four experimental signaling conditions, F+S+, F+S-, F-S+, F-S-.

Task stage	Condition Scale items	F+S+ *N* = 23	F-S+ *N* = 22	F+S- *N* = 21	F-S- *N* = 23
Presence 1: low; 5: high	Immersion	4.23 (0.79)	4.12 (075)	4.33 (0.86)	4.22 (0.52)
	Realism	3.58 (0.83)	3.28 (0.92)	3.38 (0.97)	3.36 (0.57)
	Recognition Elements	3.86 (1.01)	3.56 (0.95)	3.90 (0.83)	3.90 (0.73)
	Recognition Graph	4.18 (0.72)	3.96 (0.49)	4.09 (0.62)	4.05 (0.64)
	Total Mean/5	3.96 (0.59)	3.73 (0.49)	3.93 (0.66)	3.88 (0.37)
Cognitive Complexity Extrinsic 1: difficult; 5: easy	Ease of movements	4.54 (0.72)	4.57 (0.56)	4.33 (0.79)	4.59 (0.78)
	Ease of interaction features	4.46 (0.72)	4.53 (0.57)	4.71 (0.46)	4.62 (0.48)
	Total Mean/5	4.50 (0.45)	4.55 (0.34)	4.52 (0.46)	4.59 (0.50)
Cognitive Complexity Intrinsic 1: easy; 5: complex	Navigation complexity	1.41 (0.72)	1.67 (0.69)	1.38 (0.74)	1.72 (0.81)
	Specific knowledge	3.23 (0.73)	3.36 (0.63)	3.28 (1.00)	3.32 (0.87)
	Total Mean/5	2.32 (0.51)	2.51 (0.48)	2.33 (0.58)	2.52 (0.67)
Sickness 1: none; 5: high level	Mean/5	1.14 (0.34)	1.39 (0.47)	1.24 (0.44)	1.23 (0.42)
Enjoyment 1: low; 5: high	Mean/5	4.45 (0.72)	4.27 (0.60)	4.28 (0.56)	4.32 (0.46)

Firstly, participants all understood well the scales, and at the descriptive level, the scores observed for the four groups were fairly similar. The subjective scores for presence are high (3.73 to 3.96/5), in particular for the item relating to the specific sense of immersion (4.12 to 4.33/5). As far as extrinsic complexity is concerned, learners found their experience easy (4.50 to 4.59/5). Intrinsic complexity was scored lower, not because of the complexity of navigation, which was judged to be easy (1.41 to 1.72/5), but because of the perceived requirement for specific knowledge (3.23 to 3.36/5). While participants did not feel sickness (with low scores from 1.14 to 1.39/5), overall enjoyment was scored quite high (from 4.27 to 4.45).

Secondly, in order to explore the hypotheses, about the relation between decision performances and subjective experience of presence, perceived cognitive complexity, sickness and overall enjoyment, and especially H6, linear regressions with the homogeneity-of-slopes model were conducted for each of the five categories of measures, with the subjective score included as the continuous covariant predictor, the two signaling levels as between-subjects factors and the performance on decision 1 and decision 2 as the dependent variables. Models such as this can be used to test whether continuous predictors, as covariate moderators, have different effects at different level of categorical independent variables. For each regression, we included the interactions between the subjective factor and the signaling factors (i) tree/plant flashing and (ii) marker stones in the model. Such analyses could reveal a potential moderating effect of the perceived level of presence on decision performances.

The results of the regression analyses are presented in Table 5.

**Table 5:** Linear regression analyses for each of the five subjective scale items categories (presence, cognitive complexity-extrinsic, cognitive complexity-intrinsic, sickness and enjoyment) for decision 1 and decision 2. Main regression effects on decisions and interactions between subjective scale categories and signaling factors, respectively, flashing and marker stones. In bold, significant effects.

Scales items categories	Decision time	Main regression effect of subjective scale categories on decisions	Interactions between subjective scale categories and signaling factors: flashing and marker stones
Presence	Decision 1	**Presence: *F*(1,81) = 4.91, *p* < 0.03** ***β* = 0.21**	Pres*flash: *F*(1,81) = 0.32, *p* = 0.85 Pres*marker stones: *F*(1,81) = 0.51, *p* = 0.47
	Decision 2	**Presence: *F*(1,81) = 5.83, *p* < 0.02** ***β* = 0.26**	Pres*flashing: *F*(1,81) = 0.19, *p* = 0.66 Pres*marker stones: *F*(1,81) = 0.25, *p* = 0.56
Cognitive Complexity Extrinsic	Decision 1	Extrinsic Complexity: *F*(1,81) = 0.1, *p* = 0.98	ECompl*flash: *F*(1,81) = 0.11, *p* = 0.73 ECompl*marker stones: *F*(1,81) = 0.33, *p* = 0.56 ECompl*Flash*marker stones: *F*(1,81) = 5.00, *p* < 0.03
	Decision 2	Extrinsic complexity: *F*(1,81) = 0.01, *p* = 0.99	ECompl*flash: *F*(1,81) = 0.20, *p* = 0.66 ECompl*marker stones: *F*(1,81) = 0.95, *p* = 0.33
Cognitive Complexity Intrinsic	Decision 1	Intr-Complexity: *F*(1,81) = 0.19, *p* = 0.66	ICompl*flash: *F*(1,81) = 1.31, *p* = 0.25 ICompl*marker stones: *F*(1,81) = 0.23, *p* = 0.63
	Decision 2	Intr-Complexity: *F*(1,81) = 0.10, *p* = 0.92	ICompl*flash: *F*(1,81) = 0.71, *p* = 0.40 ICompl*marker stones: *F*(1,81) = 0.040, *p* = 0.84
Sickness	Decision 1	Sickness: *F*(1,81) = 1.07, *p* = 0.30	Sick*flash: *F*(1,81) = 0.05, *p* = 0.82 Sick*marker stones: *F*(1,81) = 3.52, *p* = 0.07
	Decision 2	Sickness: *F*(1,81) = 0.037, *p* = 0.85	Sick*flash: *F*(1,81) = 0.30, *p* = 0.58 Sick*marker stones: *F*(1,81) = 3.12, *p* = 0.08
Enjoyment	Decision 1	Enjoyment: *F*(1,81) = 1.98, *p* = 0.16	Enjoy*flash: *F*(1,81) = 2.27, *p* = 0.13 Enjoy*marker stones: *F*(1,81) = 1.21, *p* = 0.27
	Decision 2	***Enjoyment: *F*(1,81) = 3.55, *p* = 0.063, *β* = 0.19***	Enjoy*flash: *F*(1,81) = 5.92, *p* = 0.017 Enjoy*marker stones: *F*(1,81) = 0.52, *p* = 0.47

Main regression effects on decisions and interactions between subjective scale categories and signaling factors, respectively, flashing and marker stones.

**Figure 5:**
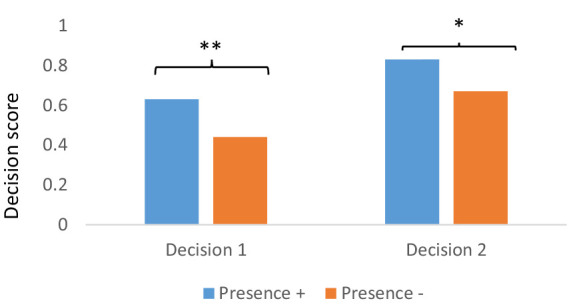
Mean performances (ratio) for decision 1 and decision 2, for participants with a high presence score (>the median = 3.87/5) and participants with a lower presence score (< the median of the group 3.87/5). Statistical *p* values: **p < 0.01, *p < 0.05.

As shown in Table 5, positive significant effects of presence on performances for decision 1 and 2 were found, as was a marginal positive effect of enjoyment in decision 2. The effect of presence on performance was not significantly moderated by signaling. Enjoyment was positively influenced by flashing for decision 2. The main result for presence is summarized in Figure 5.

In order to analyze these results, we performed a factorial ANOVA with flashing, marker stones and presence group relative to the median, e.g., presence + vs. presence –, as between-subjects factors, and decision 1 and decision 2, respectively, as dependent variable. The analysis showed a main positive (medium size) effect of presence for decision 1, *F*(1,81) = 6.18, *p* = 0.015, *ηp^2^* = 0.07, as well as for decision 2, *F*(1,81) = 5.33, *p* = 0.023, *ηp^2^* = 0.062.

In sum, the results for the relation between performances and subjective presence scales are consistent with the immersion principle developed by Makransky (2022).

## 4 Discussion-conclusion

The goal of this research was to investigate the effect of the signaling and feedback principles in an IVR learning environment. To date, very little previous research has investigated signaling (Albus and Seufert, 2022; Zhang et al., 2023) and feedback (Stenberdt and Makransky, 2023) in learning in IVR environments and, to our knowledge, no study has tested the combined use of signaling and feedback. The results of the few previous studies are consistent with the large body of research conducted in multimedia learning that have shown a small to medium positive effect of signaling on learning, comprehension and transfer (de Koning et al., 2007, 2009, 2010a,b; Boucheix and Lowe, 2010; de Koning et al., 2011; Boucheix et al., 2013; Richter et al., 2016; Schneider et al., 2018; Alpizar et al., 2020). We designed two levels of signaling in the light of the CAMIL (Makransky and Petersen, 2021), CTML (Mayer, 2009) and APM (Lowe and Boucheix, 2008, 2016) models, and because of what we have speculatively called the *“sense of proprioceptive visuospatial comprehensiveness”* in IVR relative to the potential *“all-encompassing information sensation”* of IVR (see also, Naccache, 2020).

The goal of the signals was to enhance the structuring of forest elements at the ecosystem level during information processing, mainly in terms of their spatial location in the environment.

Regarding feedback, an information text was presented together with pictures of the forest areas in an on-screen multimedia document. The goal was to prompt learners to compare the information stored in memory during the learning-by-exploration phase with the information presented in the feedback and to establish relations between these elements. Making internal comparisons might make learners aware of the presence of relevant forest elements that they may not have noticed during the exploration phase. They could then compare the different areas with each other and interpret the role of potential new elements in the light of those found during the IVR exploration.

With regard to H1 and H2, the results of this vocational education and training task, in which the participants had to make decisions regarding a human intervention having a potential impact on an ecosystem in an IVR environment, showed a positive (medium size) effect of signaling on decision performances. Signaling not only reduced the number of relevant forest elements selected (indicating that signals efficiently direct students’ attention toward the task-relevant information) but also significantly increased correct decision (decision + justification) performances.

As predicted by H3, a strong effect of informative feedback on decision 2 performances was found. This is consistent with the feedback principle in multimedia learning (Johnson and Marraffino, 2022). Additionally, and more interestingly, the interaction between signaling levels and feedback indicated that the levels of signaling had different weights at the two decision-making times, i.e., decision 1 after exploration, and decision 2 after feedback (H4).

For decision 1, the presence of marker stones (signaling soil properties, microhabitats) improved decision performance, whereas flashing (signaling tree and plant species) did not greatly affect decision performance; however, in the absence of marker stones, flashing did improve decision 1 performance. Decision performances were poorest in the no-signals condition. However, as shown in Table 2, the cumulative presence of both signaling levels did not change decision performance for this first decision compared to the marker stones-only signaling level.

For decision 2, which was given after the informative feedback, the positive effect of flashing significantly increased decision performances, and the detrimental effect of the absence of the marker stones signaling level seemed to be compensated for by the feedback. In sum, the two levels of signaling both contributed to the elaboration of high-quality mental models of the ecosystems of the forest areas at different times during training.

While our results appear to favor the use of different levels of signaling in complex immersive environments because they allow students to better organize the relevant elements in these environments, there is such thing as too much, because the accumulation of too many signals could be counterproductive and increase cognitive load. Further research is still needed on this issue.

One interesting potential explanation of the interaction between signaling levels and feedback in decision performances might involve a two-step cognitive mechanism. (i) During the learning-by-exploring phase, signaling effectively directs learners’ attention toward relevant information in such a way that the amount of task-relevant information to be stored and remembered (e.g., forest elements potentially relevant for ecosystem preservation) is reduced and more structured (compared to the no-signals condition); (ii) During the processing of the information in the feedback (multimedia document), the activation, retrieval from memory, and comparison of this selected, condensed information about the task-relevant concepts present in the feedback information could be enhanced. As a consequence, decisions are taken on the basis of higher-quality mental models of the ecosystems in the forest areas and are therefore more relevant. Of course, this explanatory hypothesis of interaction between signaling and feedback will require further empirical investigation.

As predicted by H5, the pattern of results regarding justifications was consistent with the decision performances. The two signaling levels, flashing and marker stones, had positive effects on the verbalization of true arguments focusing mainly on biodiversity, but again exerted these effects at different stages of the task, i.e., at decision 1 and decision 2. The presence of marker stones signals was more influential in decision 1, when the flashing level had only a marginal positive effect. However, the flashing level was more influential for decision 2. Furthermore, while the signaling conditions enhanced the production of more true arguments than the no-signal condition both before and, to a less extent, after feedback, the number of true arguments also increased in the no or less signaled conditions after the feedback. This trend was consistent with the effect of feedback on correct decisions. Finally, the effect of signaling on the correct justification of the selected elements was less significant in the exploration phase. This result appears logical and consistent with the idea that students were, at this stage, exploring the forest areas and then progressively building a mental model of the ecosystem of each area over time.

The relation between the complementary subjective measures of (i) presence, (ii) cognitive – extrinsic – ease of interactions, (iii) cognitive – intrinsic – task complexity, (iv) sickness, (v) enjoyment and decision performance was assessed. The main goal of the regression analyses, in which the signaling factors were included as moderators, was to identify the potential covariant relation between presence and decision performances (H6) within the framework of the immersion principle (Makransky, 2022). The main result showed a significant positive effect (medium size) of presence on decision performances for both, decision 1 and decision 2, a finding which is consistent with the immersion principle.

However, the non-significant interactions between signaling factors and presence indicated that the positive effect of presence on decision performances seemed to be unaffected by signaling (either marker stones or flashing). This is a potentially interesting result in itself, suggesting that adding guiding signals in an IVR environment does not seem to greatly disrupt the sense of presence. This should clearly be further investigated in follow-up experiments.

In sum, our main result seems consistent with the few previous studies that have investigated the signaling principle in IVR, showing its effectiveness in the same way as in multimedia screen-based presentations. Albus and Seufert (2022) and Zhang et al. (2023) tested the effect of verbal signaling, while we tested the effect of more visual cueing.

The present study also has some limitations. First of all, we did not use a typical “conventional multimedia learning lesson” but instead a more vocational learning -or training- task. This type of task is naturally of greater ecological interest, particularly in the context of recent changes in forestry training and student learning geared toward sustainable forestry management in response to global warming. However, the generalizability of the signaling principle should be also tested in more conventional IVR learning contexts.

Furthermore, the feedback provided to the students after the first decision, following the exploration phase, was limited to giving a “standard” multimedia information about the composition of the forest areas (trees, plants, moisture etc.) in static text-picture digital documents presented outside of the IVR environment. As a consequence, the feedback was not part of the IVR experience. However, more realistic feedback presented as part of the IVR experience and dynamically showing realistically the effect of the decision on the ecosystem of the forest area would be much more relevant. In the task used in the present study, this type of dynamic feedback could simulate what happens to the ecosystem once the public reception area has been set up. In particular, this should enhance the establishment of relations between the selected elements that are processed during the exploration phase, the decision and the dynamic feedback of information. These considerations will be addressed in a subsequent follow-up study.

Different levels of signaling were used. Our results suggest that different levels of signaling may have distinct effects at different levels of learning performance. However, signaling levels should be better defined and, in particular, their potential effects should be tested in more varied learning situations. It is necessary to investigate in greater detail the ways in which signals are perceptually and cognitively processed in IVR environments and how learners build relations between the signaled information and the feedback information. Direct and online measures, such as eye tracking integrated into the VR headset, could be used. This will also form part of a future experiment.

Further, the present work was conducted within the specific framework of testing multimedia learning principles (Mayer and Fiorella, 2022) in IVR environments, and in the background context of educational cognitive psychology. However, our results about the benefits of visual (and verbal) guidance techniques, and more generally about IVR environments for learning, could be used in other engineering sciences. For example, in Brain Computer Interface, IVR, as well as multimedia principles, could be investigated in order to test cost-effect balance (Mridha et al., 2021). Similarly, comparisons between IVR, Augmented Reality (AR) and Mixed Reality in learning STEM may be of great interest (Parong, 2022). Time locked attentional processes and cognitive load during IVR learning could be better assessed using precise physiological measures such as eye-tracking (Holmqvist and Andersson, 2017) and EEG (Makransky et al., 2019b) that open windows on internal cognitive processes. Such measures could be compared to the signal processing devices. Then, eye tracking and brain activity measurement data (EEG, fNIRS- functional Near Infrared Spectroscopy) could be used in the analysis of programming technologies such as LINQ (Language Integrated Query: name for a set of technologies based on the integration of query capabilities directly into the C# language) and algorithms. Finally, there are many other research avenues that could be investigated using IVR. For example, and among many other possibilities, in the domain of metacognition, it could be relevant to address how learning experience in IVR can change a person’s self-confidence or self-efficacy (de Bruin et al., 2020), or in a very different field, specific IVR software or programs could, perhaps, be developed to support vision screening.

In conclusion, this study was carried out as part of a recent line of research which consisted in systemically testing the application of multimedia principles (Mayer, 2009; Mayer and Fiorella, 2022; Makransky, 2022) to IVR environments. The present work is consistent with the small number of studies that have already been conducted in this area (Albus and Seufert, 2022; Zhang et al., 2023) and suggests that the signaling principle has significant beneficial effects in immersive environments.

## Data availability statement

The raw data supporting the conclusions of this article will be made available by the authors, without undue reservation.

## Ethics statement

The studies involving humans were approved by the Ethics Committees of the Academic Inspection of the French National Education, Bourgogne Franche-Comté region, and the Regional Direction for Digital Education (DRNE). The studies were conducted in accordance with the local legislation and institutional requirements. Written informed consent for participation in this study was provided by the participants’ legal guardians/next of kin.

## Author contributions

LP: Conceptualization, Formal analysis, Investigation, Methodology, Validation, Writing – original draft, Writing – review & editing. J-MB: Conceptualization, Data curation, Formal analysis, Funding acquisition, Investigation, Methodology, Project administration, Resources, Software, Supervision, Validation, Visualization, Writing – original draft, Writing – review & editing. LR: Resources, Software, Visualization, Writing – original draft, Writing – review & editing. VD-Z: Formal analysis, Investigation, Supervision, Writing – original draft, Writing – review & editing. J-LM: Software, Supervision, Visualization, Writing – original draft, Writing – review & editing.

## Appendix A

For each question, circle the answer that best describes you on this scale. There are no right or wrong answers ☺.

Presence

1. The graphic design is realistic and easy to interpret. (From not at all to yes, absolutely)
2. The virtual environment allowed me to feel (bodily) immersed – as if I were present there- in the experience. (From not at all to yes, absolutely)
3. The virtual environment appeared real. (From not at all to yes, absolutely)
4. It was easy for me to recognize and identify the elements of the virtual reality environment. (From not at all to yes, absolutely)

Cognitive complexity – Extrinsic

5. I was quickly able to move around and get to grips with the interface. (From not at all to yes, absolutely)
6. The features (mark a tree, access the map, etc.) were useful to me. (From not at all to yes, absolutely)

Cognitive complexity – Intrinsic

7. I lost time during exploration because of the complexity of the environment. (From not at all to yes, absolutely)
8. I had to call on my own knowledge in order to answer the problems posed by the experiment. (From not at all to yes, absolutely)

Sickness

9. I felt uncomfortable during this experiment: uncomfortable helmet, nausea, loss of orientation (From not at all to yes, absolutely)

Enjoyment

10. My appraisal of this virtual reality experience was rather: (From Highly negative to Highly positive)

Open question

11. Comments on the use of the program and virtual reality: qualities/possible defects; other:
